# Enhancement of Exposure and Reduction of Elimination for Paeoniflorin or Albiflorin via Co-Administration with Total Peony Glucosides and Hypoxic Pharmacokinetics Comparison

**DOI:** 10.3390/molecules21070874

**Published:** 2016-07-01

**Authors:** Weizhe Xu, Yan Zhao, Yi Qin, Beikang Ge, Wenwen Gong, Yingting Wu, Xiaorong Li, Yuming Zhao, Pingxiang Xu, Ming Xue

**Affiliations:** 1 Department of Pharmacology, School of Basic Medical Sciences, Capital Medical University, Beijing 100069, China; xwzccmu@126.com (W.X.); 13520339253@163.com (Y.Z.); jin_mo@live.cn (Y.Q.); gbb624@163.com (B.G.); gongww12345@126.com (W.G.); madingdaier@aliyun.com (X.L.); yumingzhao@ccmu.edu.cn (Y.Z.); syxpx88@163.com (P.X.); 2 Core Facilities Center, Capital Medical University, Beijing 100069, China; wuyt@163.com; 3 Beijing Laboratory for Biomedical Detection Technology and Instrument, Beijing 100069, China

**Keywords:** paeoniflorin, albiflorin, total peony glucosides, pharmacokinetics, interaction, hypoxic, UPLC-MS/MS

## Abstract

There is evidence suggesting that herbal extracts demonstrate greater bioactivities than their isolated constituents at an equivalent dose. This phenomenon could be attributed to the absence of interacting substances present in the extracts. By measuring the pharmacokinetic parameters of paeoniflorin (PF) and albiflorin (AF) after being orally administered to rats in isolated form, in combination with each other and within total peony glucosides (TPG), respectively, the current study aimed to identify positive pharmacokinetic interactions between components of peony radix extracts. Moreover, the pharmacokinetic profiles of PF and AF under normoxia and hypoxia were also investigated and compared. In order to achieve these goals, a highly sensitive and reproducible ultra-peformance liquid chromatography–mass spectrometry (UPLC-MS) method was developed and validated for simultaneously quantitation of PF and AF in rat plasma. This study found that compared with that of single component (PF/AF), the exposure of PF in rat plasma after combination administration or TPG administration was significantly increased, meanwhile the elimination of PF/AF was remarkably reduced. It was also noticed that AUC and *C*_max_ of PF in hypoxia rats were significantly decreased compared with that of normaxia rats, suggesting that there was a decreased exposure of PF in rats under hypoxia. The current study, for the first time, revealed the pharmacokinetic interactions between PF/AF and other constitutes in TGP and the pharmacokinetic profiles of PF and AF under hypoxia. In view of the current findings, it could be supposed that the clinical performance of total peony glucosides would be better than that of single constitute (PF/AF). The outcomes of this animal study are expected to serve as a basis for development of clinical guidelines on total peony glucosides usage.

## 1. Introduction

**Figure 1:**
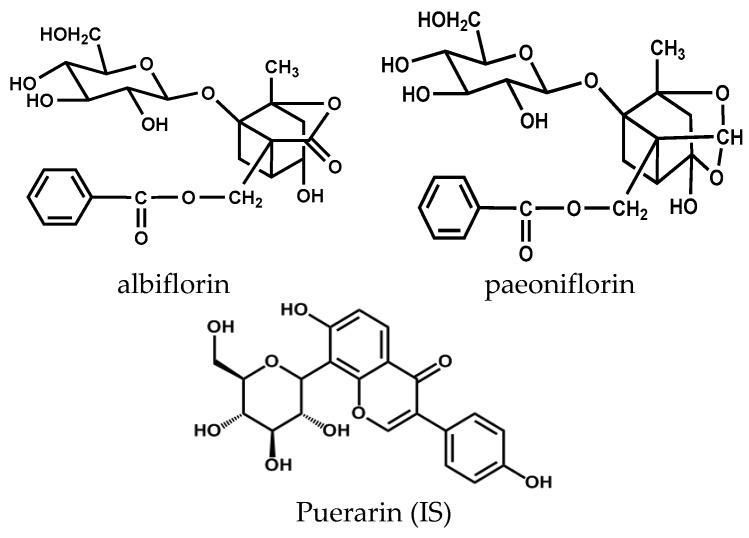
The chemical structures of albiflorin, paeoniflorin and puerarin (IS).

Peony (*Paeonialactiflora Pall*, *PLP*; family Ranunculaceae) has been widely used for treating rheumatoid arthritis, systemic lupus erythematosus, hepatitis, dysmenorrhea, muscle cramping and spasms in China, Korea, Japan and the Southeast Asian countries. The major active components of peony radix extracts (TPG) for treating these diseases are monoterpene glycosides, galloyl glucoses and phenolic compounds [1,2,3,4,5]. The ethanol/water extracts of Peony Radix are known as the total peony glucosides (TPG) that contain more than fifteen active components. Among them, paeoniflorin (PF) and albiflorin (AF) (see Figure 1), a kind of monoterpene glycoside compounds, are the most abundant ingredients and account for the pharmacological effects observed for TPG in both in vitro and in vivo studies [6,7]. These two active components are also widely applied as the effective prescriptions in Traditional Chinese Medicine (TCM) formulas for alleviating depression [8], acute myocardial infarction [9], and pain [10,11]. PF/AF exhibit wide pharmacological activities including anti-oxidation, anti-inflammatory, anti-diabetic, anti-depression and anti-hypertension effects [12,13]. Recent studies demonstrated that PF could inhibit the apoptosis of nucleus pulposus cells and activate the caspase-3 and caspase-9 via regulation of the Bcl-2 family protein expression [14]. PF was able to reduce H_2_O_2_-induced toxicity by blocking the activation of the neuroinflammatory factor NF‑κB [15]. PF could also inhibit growth of human colorectal carcinoma HT 29 cells, as well as protect against cobalt chloride-induced apoptosis of endothelial cells via the HIF-1α pathway [16]. AF, another effective monoterpene glycoside, was found to exhibit the inhibitory effects on Aβ1-40 and Aβ1-42 aggregation and neuroprotective action on hippocampal neuronal cells [17]. AF could also enhance the mitochondrial function via suppressing the antimycin A-induced oxidative damage [18]. Despite many reports about in vivo quantitative methods using LC equipped with MS to detect PF and AF [19,20], there are few reports on their in vivo simultaneous detection using UPLC-MS/MS methods. Additionally, studies involving the pharmacokinetic interactions of TPG preparations and hypoxia pharmacokinetic comparisons are even rarer.

Although TPG with its active components PF and AF has many pharmacological activities and has been widely used in clinical practice for many years, research on the possible co-administration interactions of PF, AF and TPG is insufficient. In this study, we attempted to understand the possible potential drug-drug pharmacokinetic interactions among PF, AF, which are the main active components and biomarkers in TPG preparations, and TPG. Thus, a specific and rapid ultra-performance liquid chromatography coupled with a triple quadrupole electrospray tandem mass spectrometry (UPLC-MS/MS) method was successfully developed to reveal the pharmacokinetic interaction profiles of PF, AF and TPG in rats after an oral administration of the form regimens via a single administration of PF/AF, PF co-administration with AF, PF in TPG and AF in TPG, respectively. In addition, since a large number of pharmacological studies have revealed the remarkable protective effect of PF on endothelial cells and suggested its potential application to reverse hypoxia damage [21,22], it is reasonable to monitor and compare the pharmacokinetic properties of PF and AF under both hypoxic and normoxic conditions in our current study. Our findings demonstrated that there was a higher exposure and lower elimination of PF/AF in rats after oral administration of TPG, which may partially account for the better performance of TPG extracts than that of single compound (PF/AF) in clinical practice. Thus, the outcomes of our animal study are expected to serve as a basis for the development of clinical guidelines on peony radix usage under both normoxia and hypoxia.

## 2. Results and Discussion

### 2.1. Method Development

The ZORBAX Eclipse plus C_18_ column (Agilent Technology, Palo Alto, CA, USA) was evaluated and proved to be suitable for simultaneous separation of these compounds. In order to achieve short retention times and symmetric peak shapes, several combinations of acetonitrile, methanol, formic acid and acetic acid were applied to optimize the mobile phase. It was found that methanol (A) with 0.2% formic acid in water (B) system provided an appropriate retention time, low background noise and better peak sensitivity. The mobile phase condition was 79:21 (A:B, *v*/*v*) and the flow rate was 0.4 mL/min. The electrospray ionization (ESI) source mode, the capillary temperature, fragmentation energy, and vaporizer temperature were optimized to achieve lower levels of background noise and maximum response. The compounds AF and IS were detected in positive ion mode, while PF was detected in negative ion mode, which was found to give better sensitivity for the active compounds.

### 2.2. Method Validation

#### 2.2.1. Specificity and Linearity

**Figure 2:**
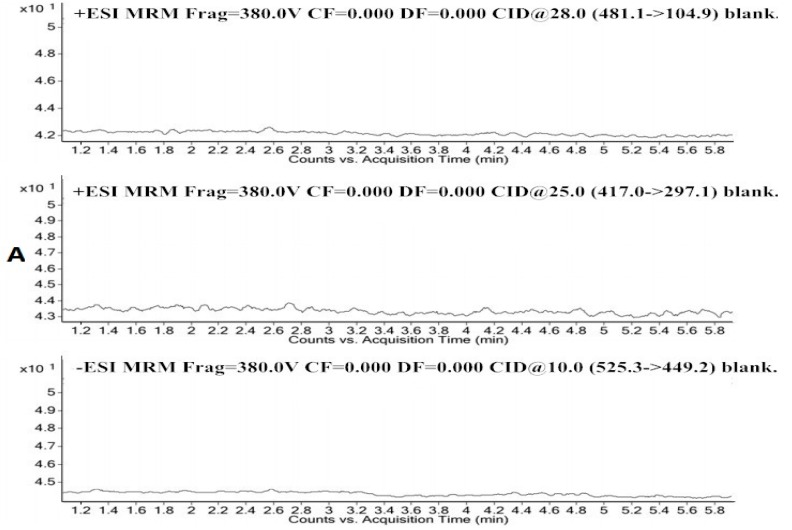

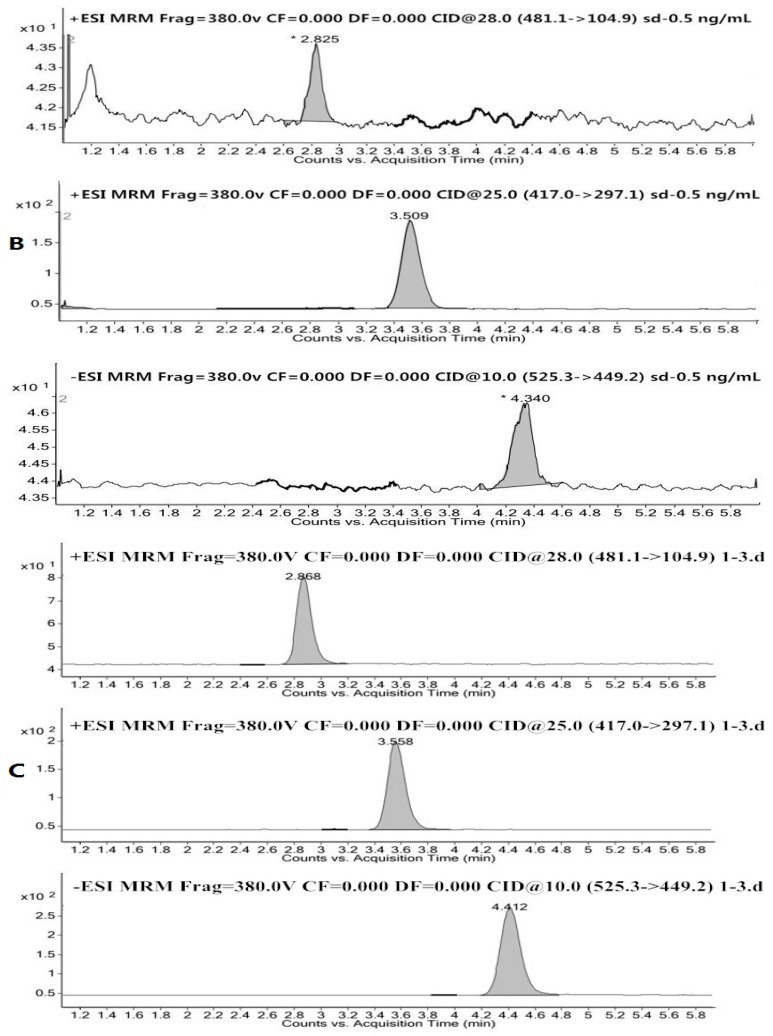
Representative MRM chromatography of a blank plasma (**A**); a blank plasma spiked with albiflorin (2.82 min), paeoniflorin (4.34 min) and the IS (3.50 min) of LLOQ (**B**); and rat plasma sample collected at 0.5 hour after administration (**C**).

The specificity was evaluated by comparing the chromatograms of blank plasma (from five different batches of rats), blank plasma spiked with PF, AF and IS, and plasma samples obtained from male Sprague-Dawley rats administrated PF, AF or TPG, respectively. The representative MRM chromatograms of the blank SD rat plasma (A), of the blank rat plasma spiked with AF and PF of the LLOQ (B), of the in vivo plasma sample obtained 0.5 h after intragastric administration of AF and PF (C) are shown in Figure 2.

The retention times of PF, IS and AF were 2.9, 3.6, and 4.4 min, respectively. No significant interference from the endogenous components has been observed at the retention times of the analytes and IS. The regression equations and correlation coefficients (r) of AF and PF were y = 0.002 + 0.007x, r^2^ = 0.999 and y = 0.003 + 0.011x, r^2^ = 0.997, respectively. The calibration curves for both PF and AF were linear in the ranges of 0.5–1000 ng/mL. The lower limit of quantification (LLOQ) can be quantitated with accuracy within 20% bias and a signal/noise ratio ≥10. The LLOQ figure from the chromatograms of the blank samples that spiked with the analytes was 0.5 ng/mL for AF and PF.

#### 2.2.2. Precision and Accuracy

The precision and accuracy of the method were assessed in plasma by performing replicate analyses of spiked samples against the calibration standards. The procedure was repeated on the same day and on several different days on the same spiked standard series. The intra-/inter-day precision and accuracy of the QC samples are presented in Table 1. Forall the analytes in QC samples, the RSD% of both intra-day and inter-day precision was below 14.6%, and the accuracy was within the range of 95.8% to 111.3%, which meets the criteria stated in the Guidance on the Bioanalytical Method Validation of the U.S.FDA.

#### 2.2.3. Recovery and Matrix Effect

The extraction recoveries of AF and PF were all between 94.4% and 114.7% (RSD < 6.5%) at three concentration of the analytes. The matrix effect ranged from 85.6% to 104.9% (RSD < 5.3%).

#### 2.2.4. Stability

The results indicated that the analytes in rat plasma were stable for 24 h after pretreatment, three freeze (−80 °C)/thaw (room temperature) cycles, and stored for 30 days at −80 °C, which demonstrated that there was a good stability for AF and PF over all steps of the determination.

### 2.3. Pharmacokinetic Interactions

#### 2.3.1. AF or Other Ingredients in TPG Enhanced Exposure and Reduced Elimination of PF in Rats

**Figure 3:**
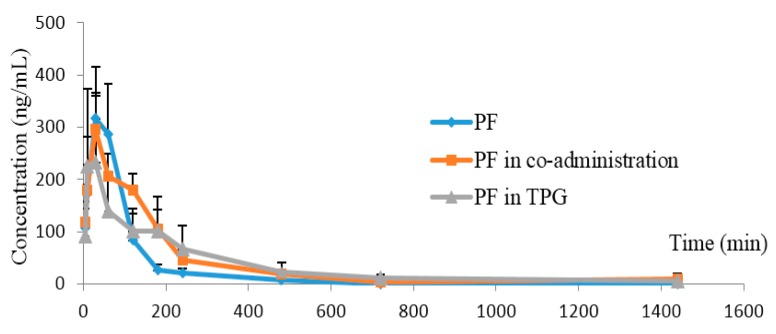
The mean plasma concentration–time profiles of paeoniflorin (PF) in rat. PF was administered orally to rats alone, PF combined with albiflorin, and PF in TPG groups. The values are the mean ± SD, *n* = 6.

The plasma concentration–time profiles of PF in rat plasma after intragastric administration of PF, PF combined with AF, and PF in TPG extracts are shown in Figure 3. The main pharmacokinetic parameters of PF in the three different groups are shown in Table 2. The results indicated that the values of the AUC and MRT of PF in the two combination groups (PF and AF group, TPG group) were significantly increased (*p* < 0.05 or *p* < 0.001) compared with the PF alone group; and the *t*_1/2_ of PF in TPG group was significantly increased (*p* < 0.01), suggesting that AF and other ingredients in TPG could enhance the exposure and reduce the elimination of PF when co-existed in rat plasma.

#### 2.3.2. Other Ingredients in TPG Reduced Elimination of AF in Rats

**Figure 4:**
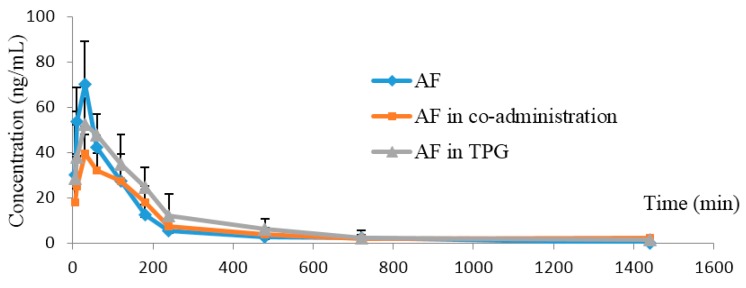
The mean plasma concentration–time profiles of albiflorin (AF) in rat plasma. AF was administered orally to rats in the alone, AF combined with paeoniflorin, and AF in TPG groups. The values are the mean ± SD, *n* = 6.

The plasma concentration–time profiles of AF in rat plasma after intragastrical administration of AF, AF combined with PF, and AF in TPG extracts are shown in Figure 4. The main pharmacokinetic parameters of AF in these three different groups are shown in Table 3. The results indicated that the values of MRT_(0→24h)_ and *t*_1/2_ of AF in TPG extracts were significantly increased (*p* < 0.05) compared with the AF alone group, suggesting that the complicated ingredients in TPG could reduce the elimination of AF when co-existed in rat plasma. On basis of our findings which identified the positive pharmacokinetic interactions among the ingredients of peony, it might be reasonable to recommend the extracts of Peony radix such as TPG, rather than single active ingredients (PF or AF), as a priority prescription in clinical practice.

Actually, interactions among the ingredients of herbal extracts are commonly recorded in literature [23,24,25]. The systematic exposure, metabolism and elimination of certain active components could be altered by the other co-ingredients present via the following mechanisms: (1) By changing the absorption of other compound, especially when the compound is a substrate of some influx or efflux transporters such as P-gp; (2) By changing the metabolism of other compounds. Some herbal constituents could compete for the same cytochrome P_450_ enzymes and thus inhibit the metabolism of each other. Moreover, many herbal ingredients are reported to change the pharmacokinetic behavior of others by altering the activities of intestinal bacteria [26,27]; (3) Alteration in blood flow rate and in renal tubular permeability could also change the elimination of some compounds. In accordance with previously published findings that indicated poor absorption and rapid elimination of PF/AF [5,20,28], the current study demonstrated that there were relatively low exposures of these two compounds in rat plasma after orally administration of isolated PF and AF respectively. The exposure of PF was nearly doubled in rat plasma after administration of TPG. This fact leads us to the speculation that the positive pharmacokinetic interactions among the multiple active components in TPG preparations may account for the improved absorption and reduced elimination of PF/AF in rodents.

### 2.4. Pharmacokinetics Comparisonunder Normoxic and Hypoxic Conditions

**Figure 5:**
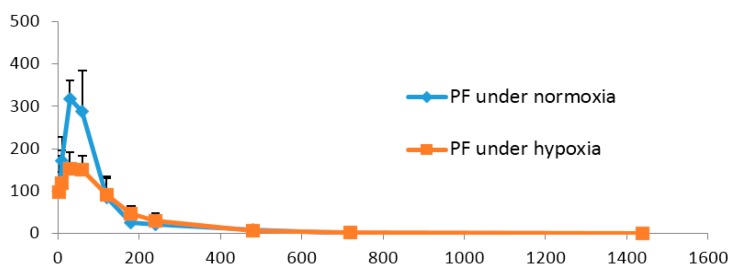
The mean plasma concentration–time profiles of paeoniflorin (PF) in rat under normoxia and hypoxia condition. PF was administered orally to rats. The values are the mean ± SD, *n* = 6.

**Figure 6:**
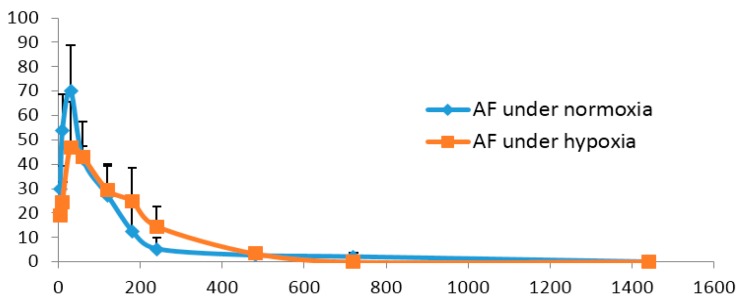
The mean plasma concentration–time profiles of albiflorin (AF) in rat under normoxia and hypoxia. The values are the mean ± SD, *n* = 6.

The mean plasma concentration-time profiles of PF and AF under normoxia and hypoxia are shown in Figure 5 and Figure 6, and the main pharmacokinetic parameters are presented in Table 4 and Table 5. It could be noticed that compared with those of normoxic rats, the values of AUC and *C*_max_ of PF in hypoxic rats were significantly decreased (*p* < 0.05 or *p* < 0.01). Hypoxia could therefore be supposed to reduce the absorption and in vivo exposure of PF in rats. However, there were no remarkable differences in the pharmacokinetic profiles of AF between normoxic and hypoxic rats. It has been reported that hypoxia, by triggering peripheral vasoconstriction, changing the actions of transporters and affecting the activities of enzymes involved in drug disposition, causes alterations in the pharmacokinetic properties of many drugs, including diazepam and dexamethasone [29,30]. Our current findings suggest that AF, which is more stable under hypoxia than PF, deserves future exploration for treating the hypoxia-induced diseases.

## 3. Experimental Section

### 3.1. Materials and Reagents

Standards of the compounds PF and AF were purchased from Shanghai Tauto Biotech Co, Ltd. (Shanghai, China). Puerarin used as the internal standard (IS) was purchased from the National Institute for Food and Drug Control (NIFDC, Beijing, China). Their purities were all in excess of 99%. The structures of these three compounds are shown in Figure 1. HPLC grade methanol was obtained from Thermo Fisher Scientific (Waltham, MA, USA). HPLC grade formic acid was purchased from the Dikma Reagent Company (Beijing, China). Water used in the experiment was double distilled. The total peonyglucosides were purchased from the Ningbo Liwah Pharmaceutical Co., Ltd (Ningbo, China). The component PF and AF used in administration was purchased from the Onaer Pharmaceutical Co., Ltd (Beijing, China). Compounds used were water solution for administration, and all other reagents and chemicals were of analytical grade and commercially available.

### 3.2. Animals

Male Sprague-Dawley (SD) rats (250 g ± 20 g) were purchased from the Animal Center of Capital Medical University (ACCMU, Beijing, China). Animals were housed in individual cages with free access to food and water in a room with the illumination (a 12 h light and dark cycle), temperature and relative humidity controlled automatically. Within five days before experiments a jugular vein in each rat under anaesthesia was cannulated by insertion of an indwelling cannula for blood sampling. After fully recovery, the rats were housed with unlimited access to food and water except for fasting 12 h before the experiment with water available. The seven groups of PF/AF and TGP were intragastrically administered to the Sprague-Dawley rats, respectively. All the animal experiments were carried out in accordance with the Guide for the Care and Use of Laboratory Animals as adopted and promulgated by the National Health Ministry of China. The protocols of animal experiments had been approved by the Animal Center of Capital Medical University.

### 3.3. UPLC-MS/MS Instruments and Conditions

The UPLC-MS/MS system consisted of a UPLC system (Agilent Technologies, Palo Alto, CA, USA) including a G4220A 1290 binary pump, a G1379A vacuum degasser, a G4226 autosampler, a G1330B 1290 thermostat and triple quadrupole mass spectrometer equipped with an electrospray ionization (ESI) source (Series 6490, Agilent Technologies). The system was controlled with the Agilent Technologies Mass Hunter Workstation B.06.00 software for data acquisition and analysis. The analytes were separated on ZORBAX Eclipse plus C_18_ column (2.1 mm × 100 mm, 3.5 μm). The mobile phase consisted of 0.2% (*v*/*v*) formic acid in water (A) and methanol (B). The mobile phase conditions were 79:21 (A:B, *v*/*v*) and the flow rate was 0.4 mL/min. The column temperature was maintained at 30 °C. The sample injection volume was 5 μL. The detection was performed on the triple quadrupole mass spectrometer equipped with electrospray source (Series 6490, Agilent Technologies). High purity nitrogen served as both the nebulizing and sheath gas. The optimized parameters in the positive or negative were as follows: capillary voltage at 3 kV, nozzle voltage at 1.5 kV, gas temperature of 200 °C and sheath gas temperature of 250 °C. The quantification was performed by multiple reaction monitoring (MRM) via precursor-to-product ion pair. AF and IS were detected in positive ion mode while PF was detected in negative ion mode. The parameters were set as follows: *m/z* 525→449 for PF (fragmentor energy, 380 eV; collision energy, 10 eV; dwell time, 80 ms); *m/z* 481→105 for AF (fragmentor energy, 380 eV; collision energy, 28 eV; dwell time, 80 ms); *m/z* 417→297 for IS (fragmentor energy, 380 eV; collision energy, 25 eV; dwell time, 80 ms).

### 3.4. Preparation of Standard Stock Solutions and Quality Control Samples

Appropriate amounts of PF and AF were precisely weighed and dissolved in methanol to produce the stock solutions. A series of mixed working solution were diluted by mobile phase to suitable concentrations of 0.5–1000 ng/mL for AF and PF. The stock solution (1 mg/mL) of IS was prepared in methanol and was further diluted with methanol to 100 ng/mL. All solutions were kept at −20 °C and brought to room temperature before use. The quality control (QC) samples used for validation procedures were similarly prepared at low, medium and high concentrations for AF and PF (1.0, 100, 1000 ng/mL) in blank plasma.

### 3.5. Plasma Sample Preparation

A 100 μL aliquot of plasma sample was added with 50 μL 1% (*v*/*v*) formic acid in water and 50 μL IS working solution (the IS, concentration of 100 ng/mL) and 300 μL acetonitrile. After vortexing for 2 min and centrifugation at 13,000× *g* for 10 min, the sample was transferred to another Axygen tube and evaporated to dryness in a rotary evaporator at 40 °C. The residue was reconstituted in 100 μL of water–methanol (79:21, *v*/*v*), then an aliquot of 5 μL was injected into the UPLC-MS/MS system for analysis.

### 3.6. UPLC-MS/MS Method Validation

#### 3.6.1. Specificity, Linearity and LLOQ

The specificity was evaluated by comparing the chromatograms of blank plasma spiked with the IS, blank plasma spiked with PF, AF and IS, and plasma samples obtained from the Male Sprague-Dawley rats administrated by PF, AF and TPG, respectively. The linearity of each calibration curve was determined by plotting the peak area ratio (y) of the analytes to IS versus the nominal concentration (x) of the analytes with a weighted (1/x^2^) least square linear regression. The lower limits of quantitation (LLOQ) of the assay was defined as the lowest concentration on the standard curve that can be quantitated with accuracy within 20% bias of the nominal concentration and the relative standard deviation (RSD) was not exceeded 20%.

#### 3.6.2. Accuracy and Precision

The accuracy and the precision of the assay for intra-day and inter-day determinations were evaluated by the analysis of three concentration levels of QC samples (*n* = 5) on the same day and on three consecutive validation days. The accuracy was expressed as the RE (%) within 85%–115% from the nominal values, and the precision as the RSD (%) within ±15% except for LLOQ, where it should be within 80%–120% for accuracy and less than 20% of precision.

#### 3.6.3. Extraction Recovery and Matrix Effect

The extraction recovery was determined by comparing of the mean concentration between the QC samples spiked with analytes in blank plasma samples and the post-extraction at the same concentration. The matrix effect was measured via comparison of the peak responses obtained from the samples where the extracted matrix was spiked with the standard solutions to those obtained from the neat standard solutions at equivalent concentrations.

#### 3.6.4. Stability

The stabilities of the analytes in the SD rat plasma were investigated using the QC sample solutions stored under different temperature conditions for different periods of time which likely to be encountered during sample storage and the analytical process. The QC samples were tested for post-treatment, three freeze-thaw cycles and long-term stabilities. The post-treatment was evaluated by placing the QC samples in the autosampler for 24 h. And for freeze-thaw cycle stability assessment, the QC samples were repeatedly freezed and thawed for three cycles at −80 °C to 20 °C. The long-term stability was carried out via placing the QC samples at −80 °C for at least 30 days.

### 3.7. in vivo Pharmacokinetic Experiment

The male Sprague-Dawley (SD) rats were randomly divided into seven groups (six rats in each group), which were housed with unlimited access to food and water, except for fasting 12 h before the experiment with water ad libitum. The hypoxic rats were exposed to a fractional concentration of 9.0% O_2_ after intragastric administration of drug for hypoxia pharmacokinetics experiments. The compound PF (80 mg/kg, normoxia and hypoxia), AF (14 mg/kg, normoxia and hypoxia), combination administration of PF (80 mg/kg) and AF (14 mg/kg), the two dosages of TPG (PF with 80 mg/kg, and AF with 14 mg/kg) were intragastrically administered to the rats, respectively. The blood sample was collected into the heparinized tubes at 0, 5, 10, 30 min, and 1, 2, 3, 4, 8, 12, 24 h before administration and post-dose. In order to get an appropriate sample for further study, 400 μL of blood was collected at each time point. Considering the physical limit of blood in rat, during the process of the pharmacokinetic experiment, each rat was received 400 μL of physiological saline at each time point after blood sampling for compensation of body fluid loss, and the blood loss was also corrected at definite degree. During the experimental process or at the end of the experiments, the physiological states of the rats were normal. The plasma samples were centrifuged at 2000× *g* for 10 min at 4 °C and then they were transferred to another Axygen tube and stored at −80 °C until analysis.

### 3.8. Data Processing and Comparison

The main pharmacokinetic parameters of PF and AF were processed and calculated by the DAS 2.1 software. One way ANOVA *t*-test was used to compare the differences of the major pharmacokinetic parameters among multiple groups. Independent-samples *t*-test was used to compare normoxic and hypoxic groups. The differences was considered statistically significant when *p* < 0.05 or *p* < 0.01.

## 4. Conclusions

A UPLC-ESI-MS/MS method has been developed for the simultaneous determination of PF and AF from PF, AF and TPG preparations in rat plasma. The performance criteria for the precision and accuracy, recovery and matrix effect, sensitivity, linearity and stability have been assessed and proved to meet the FDA’s requirements. The results indicated that this method could be successfully used for studying both the pharmacokinetic interactions of PF, AF and TPG after intragastric administration to rats, and the pharmacokinetic comparison of PF and AF between hypoxic and normoxic rats. It is noteworthy that both PF and AF showed slower elimination rates when combined with TPG, suggesting that the in vivo PF and AF levels were significantly increased based on the higher systematic exposures and slower drug elimination. The pharmacokinetic parameters of PF under hypoxia indicated that the systematic exposure was markedly decreased. The different pharmacokinetic behaviors implied that the multiple active components in TPG played an important role in the potential drug–drug interactions. Such interactions enhanced the systematic exposure and reduced the elimination of PF and AF. The pharmacokinetic interactions could shed light on the safety and efficacy of the herbal medicine, and may guide the clinical usage.

## Figures and Tables

**Table 1:** Intra-day and inter-day precision and accuracy data of the analytes in rat plasma.

Compounds	Added (ng/mL)	Intra-Day (*n* = 5)		Inter-Day (*n* = 15)	
		Accuracy (%)	Precision (RSD%)	Accuracy (%)	Precision (RSD%)
AF	1.0	96.9	13.0	108.4	14.6
	100	97.3	1.7	99.8	6.7
	1000	105.9	1.3	95.8	9.3
PF	1.0	98.8	12.3	97.8	14.2
	100	108.2	5.3	111.3	5.2
	1000	105.4	6.6	104.8	5.2

**Table 2:** The main pharmacokinetic parameters of PF with three different forms in rat plasma after intragastrical administration (*n* = 6).

Parameters (Unit)	PF Alone	PF Combined with AF	PF in TPG
AUC_(0→24 h)_ (ng/mL·min )	35,702 ± 6355.8	51,907 ± 13,552 *	54,128 ± 18,416 *
MRT_(0→24 h)_ (min)	109.6 ± 25.3	199.9 ± 97.4 **	288.8 ± 44.7 ***
*t*_1/2_ (min)	108.6 ± 37.8	97.9 ± 39.9	234.6 ± 96.2 **
*t*_max_ (min)	35.0 ± 12.2	41.7 ± 39.2	23.3 ± 10.3
*C*_max_ (ng/mL)	332.7 ± 63.6	319.8 ± 120.9	290.9 ± 141.1
CL (L·kg/min)	2.3 ± 0.4	1.6 ± 0.4	1.7 ± 0.6

Data were given as the mean ± SD; * *p* < 0.05, ** *p* < 0.01 and *** *p* < 0.001 showed significantly different compared with the PF alone group.

**Table 3:** The main pharmacokinetic parameters of AF with three different forms in rat plasma after intragastric administration (*n* = 6).

Parameters (Unit)	AF Alone	AF Combined with PF	AF in TPG
AUC_(0→24 h)_ (ng/mL·min)	8527.2 ± 2194.3	7754.7 ± 1897.5	11,777 ± 3419.4
MRT_(0→24 h)_ (min)	135.0 ± 29.3	178.2 ± 93.2	225.8 ± 32.1 *
*t*_1/2_ (min)	119.6 ± 29.7	130.1 ± 58.7	215.7 ± 91.9 *
*t*_max_ (min)	26.7 ± 8.2	41.7 ± 39.2	31.7 ± 16.0
*C*_max_ (ng/mL)	70.3 ± 19.1	42.4 ± 18.7 **	56.9 ± 12.9
CL (L·kg/min)	1.7 ± 0.5	1.8 ± 0.6	1.2 ± 0.4

Data were given as the mean ± SD; * *p* < 0.05 and ** *p* < 0.01 showed significantly different compared with the AF alone group.

**Table 4:** The main pharmacokinetic parameters of PF under normoxia/hypoxia in rat plasma after a single intragastrical administration (*n* = 6).

Parameters (Unit)	PF under Normoxia	PF under Hypoxia
AUC_(0→24 h)_ (ng/mL·min)	35,702± 6355	26,600 ± 4729 *
MRT_(0→24 h)_ (min)	109.6 ± 25.3	127.2 ± 35.3
*t*_1/2_ (min)	108.6 ± 37.8	111.6 ± 28.5
*t*_max_ (min)	35.0 ± 12.2	50.8 ± 39.8
*C*_max_ (ng/mL)	332.7 ± 63.6	176.9 ± 55.1 **
CL (L·kg/min)	2.3 ± 0.4	3.05 ± 0.54

Data were given as the mean ± SD; * *p* < 0.05 and ** *p* < 0.01 showed significant difference between the normoxia and hypoxia groups.

**Table 5:** The main pharmacokinetic parameters of AF under normoxia/hypoxia in rat plasma after a single intragastrical administration (*n* = 6).

Parameters (Unit)	AF under Normoxia	AF under Hypoxia
AUC_(0→24 h)_ (ng/mL·min)	8527.2 ± 2194	8299.0 ± 2202
MRT_(0→24 h)_ (min)	135.0 ± 29.3	129.5 ± 24.9
*t*_1/2_ (min)	119.6 ± 29.7	131.5 ± 57.5
*t*_max_ (min)	26.7 ± 8.2	35.0 ± 12.3
*C*_max_ (ng/mL)	70.3 ± 19.1	49.1 ± 16.2
CL (L·kg/min)	1.7 ± 0.5	1.6 ± 0.7
